# Killing Glioblastoma Cells with Glycosylated Indolocarbazole-Based Derivative LCS1269: A Potential Crosstalk Between Micronuclei Formation and the Concurrent Induction of Apoptosis, Necroptosis, and Pyroptosis

**DOI:** 10.3390/ph19040535

**Published:** 2026-03-26

**Authors:** Nikolay Kalitin, Alexander Masyutin, Maria Erokhina, Ekaterina Savchenko, Nadezhda Samoylenkova, Aida Karamysheva, Galina Pavlova

**Affiliations:** 1 Laboratory of Predictors of Sensitivity to Antitumor Therapy, N.N. Blokhin National Medical Research Center of Oncology, 115478 Moscow, Russia; aikaram@yandex.ru; 2 Department of Cell Biology and Histology, Faculty of Biology, Lomonosov Moscow State University, 119234 Moscow, Russia; imber.acidis@gmail.com (A.M.); masha.erokhina@gmail.com (M.E.); 3 Laboratory of Molecular and Cellular Neurogenetics, N.N. Burdenko National Medical Research Center of Neurosurgery, 125047 Moscow, Russia; savhenko61@mail.ru (E.S.); samoylenkova.n@gmail.com (N.S.); lkorochkin@mail.ru (G.P.); 4 Laboratory of Neurogenetics and Developmental Genetics, Institute of Higher Nervous Activity and Neurophysiology of RAS, 117485 Moscow, Russia

**Keywords:** glycosylated indolocarbazoles, LCS1269, apoptosis, necroptosis, pyroptosis, PANoptosis, micronuclei

## Abstract

**Background/Objectives**: Glioblastoma multiforme (GBM) is the most infiltrative, treatment-resistant, and deadly brain tumor in adults. Given the extremely malignant phenotype of the GBM cells, the high intratumoral heterogeneity, and the limited efficacy of the vast majority of chemotherapeutics due to the restrictive nature of the blood–brain barrier, GBM remains largely incurable. **Methods**: Utilizing the U87, U251, and T98G GBM cell lines, diverse in vitro approaches (Western blotting, quantitative real-time PCR, flow cytometry, immunofluorescence, Luc-reporter analysis, microscopic examination, and scanning electron microscopy), and pharmacological inhibition, we investigated for the first time the cell death decisions in the GBM cells in response to the LCS1269 treatment. **Results**: We showed that LCS1269 collapsed the mitochondrial potential and triggered both intrinsic and extrinsic apoptosis. Importantly, our findings demonstrated that LCS1269-mediated apoptosis was paralleled by an induction of both MLKL-dependent necroptosis and caspase-3/GSDME-dependent pyroptosis. Using a combination of specific inhibitors, we further demonstrated that apoptosis, necroptosis, and pyroptosis provoked by LCS1269 occur simultaneously and orchestrate a peculiar form of programmed cell death, which is known as PANoptosis. We subsequently found that LCS1269-induced PANoptosis may be initiated either through the RIPK1-PANoptosome alone or through the integrated ZBP1-, AIM2-, and RIPK1-PANoptosomes. Additionally, we revealed that LCS1269-mediated PANoptosis may be closely related to micronuclei formation. **Conclusions**: Taken together, our results confirm that LCS1269 is a promising anti-glioblastoma agent that is capable of effectively promoting GBM cell death via PANoptosis.

## 1. Introduction

Glioblastoma multiforme (GBM), an aggressive primary brain tumor, is stratified as the highest grade (IV) on the malignancy scale according to the 2021 World Health Organization Classification of Tumors of the Central Nervous System (WHO CNS5) [1]. Currently, conventional therapeutic approaches, such as surgical tumor resection, radiotherapy, and chemotherapy, which are used as the standard-of-care for the treatment of GBM, are only partially effective. This limited efficacy is due to the diffuse nature of GBMs, the rapid proliferation of GBM cells, the fast development of drug-resistant clones, and the poor penetration of therapeutics through the blood–brain barrier [2]. Despite advances in treatment, including various strategies of gene, cell, and immune therapies, GBM remains largely intractable, with a median survival of approximately 15 months [3,4]. In accordance with this, new therapeutic strategies aimed at overcoming the high recurrence rate and poor prognosis of GBM are urgently needed.

Programmed cell death (PCD) is a pivotal process for controlling tissue development and maintaining homeostasis in multicellular organisms [5]. Moreover, several evolutionarily conserved and tightly regulated forms of PCD, such as apoptosis, necroptosis, pyroptosis, ferroptosis, and autophagy, are very important for developing effective antitumor therapy and the discovery of anticancer drugs [6]. Although apoptosis, necroptosis, and pyroptosis constitute distinct signaling pathways, increasing evidence demonstrates that there is extensive crosstalk among them. This led to the concept of PANoptosis, a new type of PCD that was proposed in 2019 by Malireddi et al. [7]. PANoptosis is a novel inflammatory mode of PCD that integrates the key features of pyroptosis, apoptosis, and necroptosis, corresponding to the “P,” “A,” and “N” in the PANoptosis term, respectively [8]. This lytic form of PCD can be triggered by severe exogenous or endogenous perturbations that are detected by innate immune sensors, primarily the Z-DNA-binding protein 1 (ZBP1), which is absent in melanoma 2 (AIM2). It is executed by caspases and receptor-interacting protein kinases (RIPKs) and culminates in the assembly of a PANoptosome complex [9]. The terminal phase of PANoptosis involves a pore formation that is mediated by different gasdermins, MLKL (mixed-lineage kinase domain-like protein), and potentially other, yet to be identified molecules that may be cleaved by the caspases [10].

LCS1269 belongs to a large class of natural and synthetic heterocyclic aromatic substances known as glycosylated indolocarbazoles, in which an indolocarbazole scaffold is linked to a carbohydrate moiety via one or two N-glycosidic bonds [11]. Among glycosylated indolocarbazoles, several derivatives have been examined for anticancer therapy both in vitro and in clinical trials [11,12]. However, only one glycosylated indolocarbazole compound, midostaurin (N-benzoyl staurosporine), has been approved by the FDA as a drug for treating patients with FLT3-mutated acute myeloid leukemia [13].

In the present study, we investigated for the first time the specific modes of PCD that are triggered in the GBM cells upon treatment with LCS1269. We also aimed to clarify the distinct molecular mechanisms underlying its anti-glioblastoma effects.

## 2. Results

### 2.1. LCS1269 Provokes a Potential Mitochondrial Membrane Collapse and Promotes Both Intrinsic and Extrinsic Apoptotic Pathway Activation

**Figure 1:**
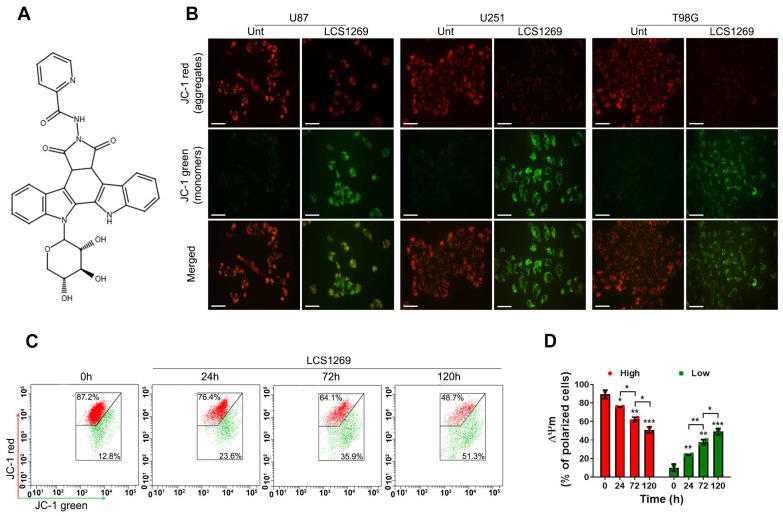
LCS1269 reduces the mitochondrial membrane potential. (**A**) The chemical structure of LCS1269. (**B**) The fluorescence intensity of JC-1 aggregates (red) and JC-1 monomers (green) in U87, U251, and T98G cells that were exposed to LCS1269 (2.5 µM) for 24 h (magnification, 400×). The scale bar = 25 µm. (**C**) LCS1269 (2.5 µM) mediates the MMP depolarization (ΔΨm) in U87 cells in a time-dependent manner. (**D**) The time course of a ΔΨm decrease is shown in the associated bar charts. The data are presented as mean ± SD of at least three separate experiments. * *p* < 0.05, ** *p* < 0.01, and *** *p* < 0.001.

Earlier, we found that LCS1269 reduced the growth of the GBM cells in a different way. IC50 values for U87, U251, and T98G cells were 14.33 ± 2.52 µM, 0.70 ± 0.08 µM and 2.83 ± 0.93 µM, respectively [14]. Therefore, effective concentrations of LCS1269 ranging from 0.5 to 2.5 µM were selected for the present study. As shown in Figure 1B, treatment with LCS1269 for 24 h strikingly reduced the mitochondrial membrane potential (MMP) in all of the tested GBM cells, with a particularly pronounced effect in the U251 cells. Furthermore, this decrease in the MMP was found to be time-dependent (Figure 1C,D).

**Figure 2:**
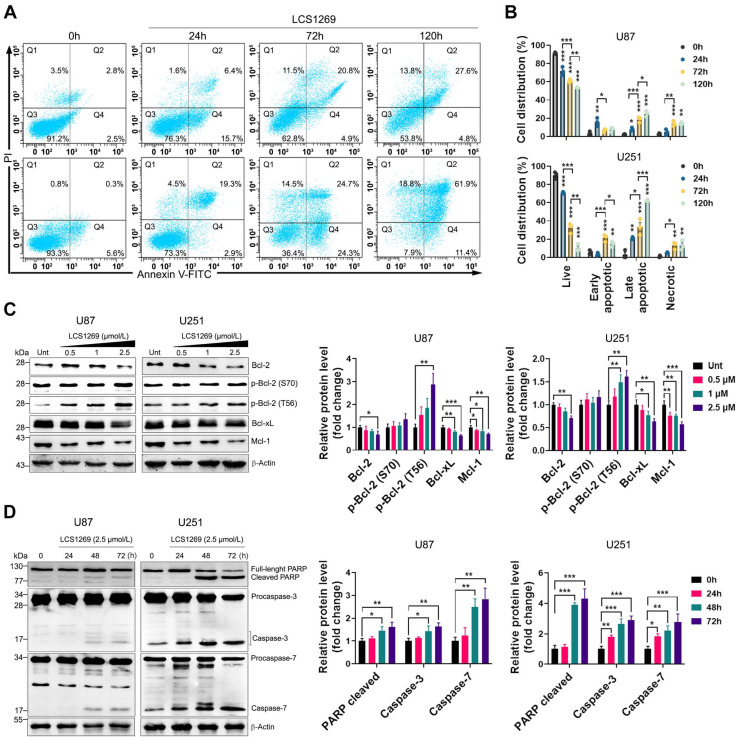
LCS1269 promotes intrinsic apoptosis. (**A**) The representative flow cytometry graphs displaying necrotic (Q1), late apoptotic (Q2), live (Q3), and early apoptotic (Q4) cell populations in U87 (**upper**) and U251 (**lower**) cells that were exposed to LCS1269 (2.5 µM) for the indicated times. (**B**) The quantification of Q1, Q2, Q3, and Q4 populations in the U87 and U251 cells that were treated in the absence or presence of LCS1269 (2.5 µM) for the indicated times. (**C**) The immunoblotting analysis of anti-apoptotic protein expression in the U87 and U251 cells that were treated with LCS1269 at the indicated concentrations for 24 h (**left**). The bar charts demonstrate the results of the relative band density of the investigated proteins that were normalized to β-Actin (**right**). (**D**) The representative images showing the abundance of pro-apoptotic proteins in the U87 and U251 cells at the indicated time points after the LCS1269 treatment (**left**). The bar charts demonstrate the results of the relative band density of the specific protein contents that were normalized to β-Actin (**right**). β-Actin was used as a loading control. The data are presented as mean ± SD of at least three separate experiments. * *p* < 0.05, ** *p* < 0.01, and *** *p* < 0.001.

It is well known that a strong MMP depolarization may cause such a type of PCD as apoptosis [15]. Our results confirmed that LCS1269 triggered a significant increase of both early and late apoptotic cells in a time-dependent manner, most obviously in the U251 cell line (Figure 2A,B).

**Figure 3:**
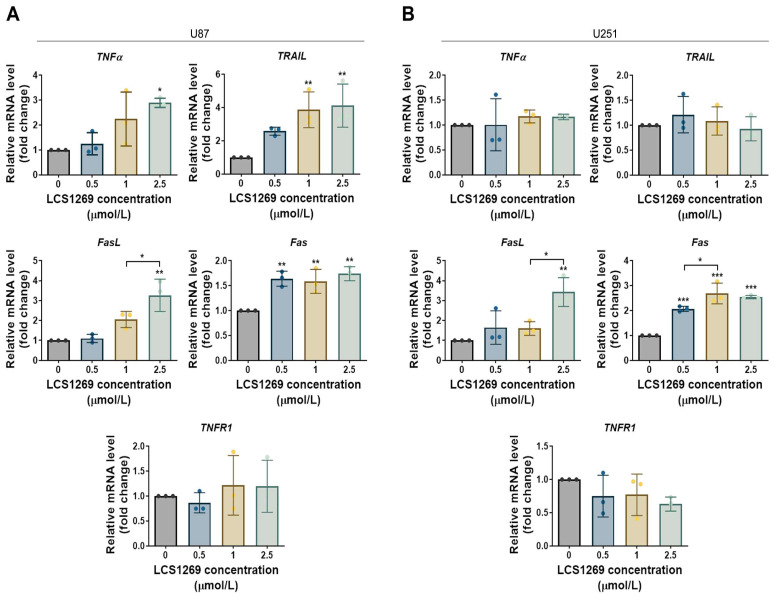
LCS1269 augments the expression of the crucial genes of the extrinsic apoptotic pathway. (**A**,**B**) The mRNA levels of the key genes within the extrinsic apoptotic pathway in U87 and U251 that were exposed to increasing concentrations of LCS1269 for 24 h were measured using qRT-PCR. The relative expression for the tested genes was normalized to the housekeeping gene *YWHAZ* and was quantified by the 2^−ΔΔCt^ method. The data are presented as mean ± SD of at least three separate experiments. * *p* < 0.05, ** *p* < 0.01, and *** *p* < 0.001.

Apoptosis is thought to be executed mainly by means of two distinct, but intersecting, signaling cascades and intrinsic and extrinsic pathways [16]. In our experiments, the MMP depolarization and apoptosis induction in the GBM cells were accompanied by the concurrent downregulation of the total levels of Bcl-2, Bcl-xL, and Mcl-1, which are the pivotal anti-apoptotic regulators of the intrinsic pathway (Figure 2C). As can be seen from Figure 2D, the LCS1269 treatment led to a pronounced activation of the key apoptotic effector proteins, caspases three and seven. This resulted in the cleavage and consequent inactivation of the essential DNA repair enzyme poly (ADP-ribose) polymerase (PARP). Importantly, the exposure to LCS1269 resulted in an overexpression of both ligand *FasL* and its receptor *Fas*, which are key components of the extrinsic apoptosis pathway in both U87 and U251 cell lines (Figure 3A,B).

Consistently, we demonstrated that LCS1269 caused a collapse in the MMP and induced both intrinsic and extrinsic apoptotic pathways.

### 2.2. Lytic Cell Death Mediated by LCS1269 Is Partially Realized Through MLKL-Dependent Necroptosis Followed by NF-κB Activation

**Figure 4:**
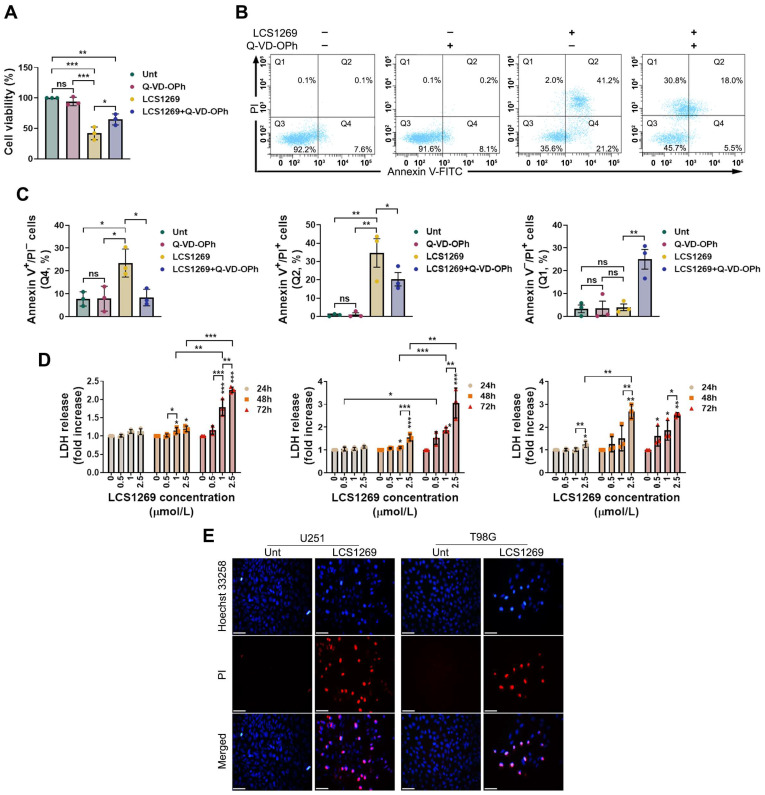
LCS1269 triggers lytic cell death. (**A**) The MTT viability assay of T98G cells that were treated with or without LCS1269 (2.5 µM) for 72 h shows that pre-treatment (for 1 h) with the pan-caspase inhibitor Q-VD-OPh (25 µM) partially prevents LCS1269 cytotoxicity. (**B**) The flow cytometry analysis of the LCS1269-exposed T98G cells that were pre-treated with or without Q-VD-OPh (25 µM) for 1 h, followed by Annexin V-FITC/PI double staining. (**C**) The quantification of necrotic (Q1), late apoptotic (Q2), live (Q3), and early apoptotic (Q4) cell populations. (**D**) LCS1269 induces the release of lactate dehydrogenase (LDH) from U87 (**left**), U251 (**middle**), and T98G (**right**) cells in a dose- and time-dependent manner. (**E**) The Hoechst 33258 plus propidium iodide (PI) staining of U251 and T98G cells that either were or were not exposed to LCS1269 (2.5 µM) for 48 h (magnification, 400×). The scale bar = 25 µm. The data are expressed as mean ± SD of at least three separate experiments. * *p* < 0.05, ** *p* < 0.01, *** *p* < 0.001, and ns—not significant.

Since LCS1269 exposure gradually augmented the percentage of the Annexin-V^+^/PI^+^ positive GBM cells, we next investigated whether the LCS1269 treatment could also trigger the forms of PCD that are characterized by a loss of plasma membrane integrity. Indeed, the pre-treatment of T98G cells with the pan-caspase inhibitor Q-VD-OPh partially rescued the cell viability following LCS1269 exposure (Figure 4A). At the same time, we verified that the Q-VD-OPh pre-treatment significantly reduced the proportions of both the Annexin-V^+^/PI^−^ (with relatively intact membranes) and the Annexin-V^+^/PI^+^ (with disrupted cell membranes) cells, while the percentage of necrotic cells (Annexin-V^−^/PI^+^) sharply increased (Figure 4B,C).

Moreover, these findings were consistent with the results of both the LDH leakage and the Hoechst 33258/PI double staining assays, which indicated the induction of lytic cell death upon the LCS1269 treatment (Figure 4D,E). Altogether, the pan-caspase inhibitor Q-VD-OPh marginally restored the LCS1269-mediated GBM cell death, suggesting the involvement of additional, caspase-independent lytic cell death mechanisms. In fact, the LCS1269 treatment caused the overexpression of both *FasL* and its receptor *Fas* (Figure 3A,B), which was associated with a progressive elevation of necrotic cells (Figure 2A,B). Additionally, pre-treatment with Q-VD-OPh diminished the caspase-dependent PCD, but concomitantly contributed to an increase in the proportion of necrotic cells (Figure 4B,C).

**Figure 5:**
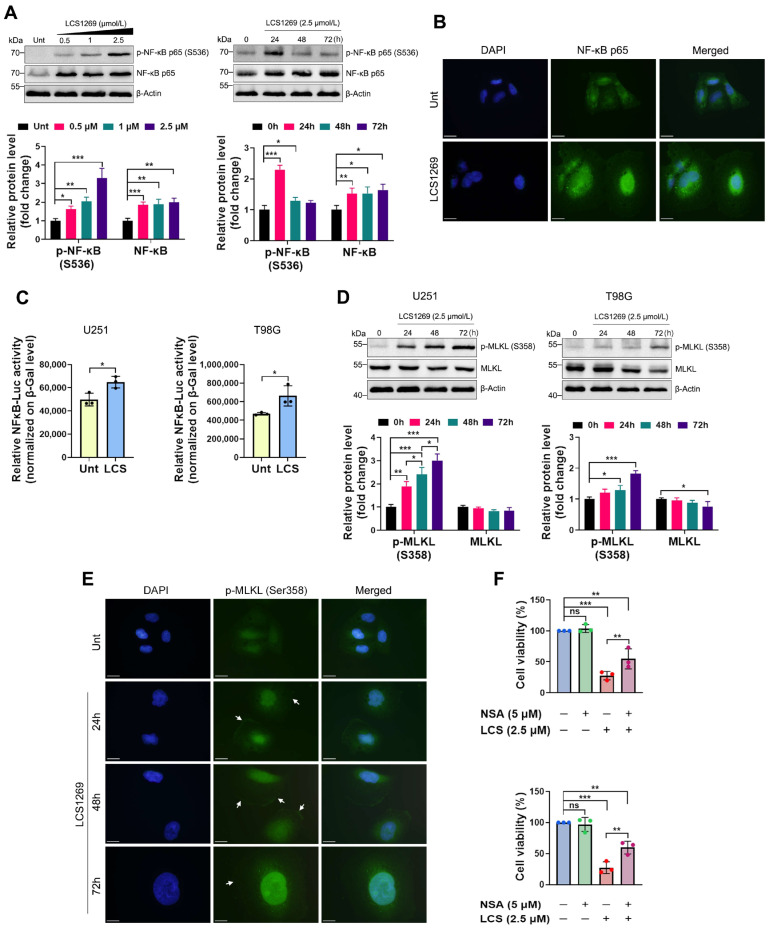
LCS1269 induces necroptosis followed by NF-κB activation. (**A**) The representative images (**upper**) show the protein levels of p-NF-κB p65 (S536) and the total NF-κB p65 in the U251 cells that were treated with various concentrations of LCS1269 for 24 h (**left**) or at the indicated time points (**right**). The bar plots demonstrate the results of the relative band density of p-NF-κB p65 (S536) and the total NF-κB p65 that was normalized to β-Actin (**lower**). (**B**) The representative images showing the nuclear–cytoplasmic distribution of the total NF-κB p65 in the U251 cells cultured in the absence or presence of LCS1269 (2.5 µM) for 24 h. DAPI was used to label the cell nuclei (magnification, 1000×). The scale bar = 10 µm. (**C**) The reporter assay of NF-κB transcriptional activity in the U251 and T98G cells treated with or without LCS1269 (2.5 µM) for 24 h. The relative NF-κB-Luc activity was calculated in arbitrary units as the ratio of the detected luciferase activity to β-galactosidase activity (β-Gal). (**D**) The Western blotting analysis of p-MLKL (S358) and the total MLKL in the U251 and T98G cells at the indicated time points (**upper**). The bar plots show the results of the relative band density of p-MLKL (S358) and the total MLKL that was normalized to β-Actin (**lower**). (**E**) The representative immunofluorescence images of p-MLKL (S358) distribution in the U251 cells that either were or were not exposed to LCS1269 (2.5 µM) for 24 h, 48 h, and 72 h (white arrows designate the cell membrane localization of p-MLKL (S358)). DAPI was used to counterstain the cell nuclei (magnification, 1000×). The scale bar = 10 µm. (**F**) The MTT assay of the U251 (**upper**) and T98G (**lower**) cells that were pre-treated with the selective MLKL inhibitor necrosulfonamide (NSA) for 1 h and then exposed to LCS1269 for 72 h. β-Actin was used as a loading control. The data are expressed as mean ± SD of at least three separate experiments. * *p* < 0.05, ** *p* < 0.01, *** *p* < 0.001, and ns—not significant.

As shown in Figure 5A, the LCS1269 treatment induced a substantial upregulation of total NF-κB levels, along with a simultaneous dose-dependent increase in its phosphorylated form at Ser536. Moreover, LCS1269 not only boosted the NF-κB expression but also promoted its nuclear translocation and activated an NF-κB-Luc reporter (Figure 5B,C).

To ensure that LCS1269 might participate in the necroptosis induction, the expression of pseudokinase MLKL as a principal necroptotic executor was investigated. The LCS1269 treatment significantly enhanced the expression level of p-MLKL (S358) in both U251 and T98G in a time-dependent manner (Figure 5D). Moreover, we revealed that LCS1269 not only upregulated p-MLKL (S358) but also prominently increased its membrane-bound fraction after 24–72 h of exposure (Figure 5E, white arrows). To further confirm MLKL’s role in the LCS1269-mediated necroptosis, we used necrosulfonamide (NSA), which is a selective MLKL inhibitor. As shown in Figure 5F, pre-treatment with NSA significantly restored cell viability in both U251 and T98G cells compared to treatment with LCS1269 alone.

Thus, our findings indicate that LCS1269-induced lytic cell death is mediated, at least in part, by MLKL-dependent necroptosis.

### 2.3. LCS1269 Is Able to Trigger Both Robust Pyroptosis and Marginal Pyroptosis-like Features in the GBM Cell Lines via a Caspase-3/GSDME Axis

We indicated above that the treatment of the GBM cells with LCS1269 led to the activation of NF-κB as one of the most important nodes, orchestrating diverse pro-inflammatory signaling cascades (Figure 5A–C). Therefore, we speculated that the LCS1269 treatment could also contribute to pyroptosis induction, concomitantly with apoptosis and necroptosis.

**Figure 6:**
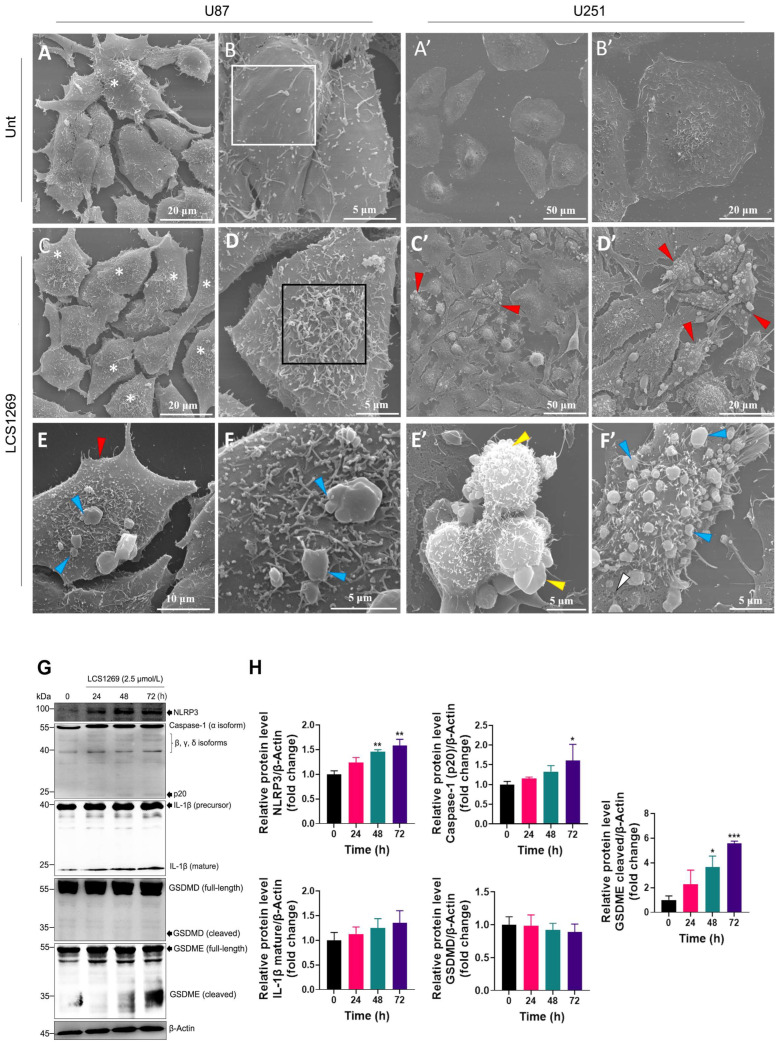
LCS1269 causes pyroptosis via the caspase-3/GSDME axis in the GBM cells. (**A**,**A’**–**F**,**F’**) The U87 and U251 cells were treated with or without LCS1269 (2.5 µM) for 48 h, and morphological features of pyroptosis were observed by scanning electron microscopy (the white frame designates an enlarged fragment on the cell surface containing few filopodia (finger-like cellular protrusions), whereas the black frame indicates an enlarged fragment on the cell surface with numerous filopodia; the white asterisks show cells with more than 70 filopodia on the cell surface; the red arrowheads annotate cells that differ from the control (untreated) ones; the blue arrowheads delineate pyroptotic bubble-like protrusions up to several microns in size; the yellow arrowheads mark apoptotic blebs; and the white arrowheads point to the plasma membrane ruptures (pores)). (**G**) The protein levels of NLRP3, caspase-1, IL-1β, GSDMD, and GSDME in the U251 cells that were treated with or without LCS1269 at the indicated time points. β-Actin was used as a loading control. (**H**) The histograms show the densitometric analysis of the band intensity for specific proteins that were normalized to β-Actin. The data are expressed as mean ± SD from at least three separate experiments. * *p* < 0.05, ** *p* < 0.01, and *** *p* < 0.001.

With the aid of scanning electron microscopy (SEM), we first investigated the changes in the plasma membrane surface in the GBM cells that were treated with or without LCS1269 (2.5 µM) for 48 h. The SEM observations showed that the U87 and U251 cells responded differently to the LCS1269 treatment. Unlike untreated cells (Figure 6A,B), U87 cells treated with LCS1269 (2.5 µM for 48 h) did not exhibit a considerable change in size (Figure 6C,D). However, after the exposure to LCS1269, non-spherical cells with bubble-like protrusions were observed, which may be a sign of pyroptosis (Figure 6E,F). The U251 cells responded to the drug more significantly than the control (Figure 6A’,B’), with numerous cells displaying an abnormal morphology (Figure 6C’,D’, red arrowheads). Among these cells, both apoptotic (spherical cells with signs of apoptotic body formation (Figure 6E’, yellow arrowheads)) and pyroptotic cells were detected (Figure 6F’, blue arrowheads). The latter retained their flattened state while forming pyroptotic bubbles. Also, in these cells, unlike U87, we observed ruptures of the plasma membrane, which may indicate later stages of pyroptosis (Figure 6F’, white arrowhead). Thus, the U251 cells demonstrated a greater sensitivity to the LCS1269 treatment and were more prone to pyroptosis induction.

Further, we assessed the expression of distinct markers that could indicate the onset of pyroptosis. As shown in Figure 6G,H, LCS1269 significantly upregulated the expression of the NLR Family Pyrin Domain Containing 3 (NLRP3) starting from 48 h post-treatment. Moreover, we revealed that the LCS1269 treatment led to a weak but significant upregulation of the caspase-1 catalytic subunit, p20. Expectedly, the expression of the mature form of IL-1β was slightly and not significantly affected. Importantly, the LCS1269 treatment did not lead to the cleavage of the gasdermin D (GSDMD) precursor or the release of its activated, cleaved form, which can form pores in the cell membrane, triggering pyroptosis. However, the treatment with LCS1269 greatly upregulated the cleaved form of another gasdermin, GSDME, in a time-dependent manner.

To dissect whether pyroptosis that was mediated by the LCS1269 treatment is triggered via the caspase-3/GSDME axis, we further used breast adenocarcinoma MCF-7 cells. It is well established that the MCF-7 cells lack the expression of both caspase-3 and GSDME due to a deletion within exon 3 of the *CASP3* gene and the promoter hypermethylation of the *DFNA5* gene (coding the GSDME protein), respectively [17,18,19]. These published data are consistent with our results, as we also failed to detect the expression of either caspase-3 or GSDME, whereas caspase-1 and GSDMD were expressed in the MCF-7 cells (Figure S1A). As anticipated, no morphological features related to pyroptosis induction were observed in the caspase-3/GSDME-deficient MCF-7 cells after the LCS1269 treatment compared to untreated cells (Figure S1B–G).

Based on these results, we reasoned that the LCS1269-mediated pyroptosis in the GBM cells is likely coordinated through the caspase-3/GSDME axis rather than the caspase-1/GSDMD pathway.

### 2.4. LCS1269 Mediates the Differential Regulation of Gene Expression of Either RIPK1 Only or the Integrated ZBP1-, AIM2-, and RIPK1-PANoptosome Components

**Figure 7:**
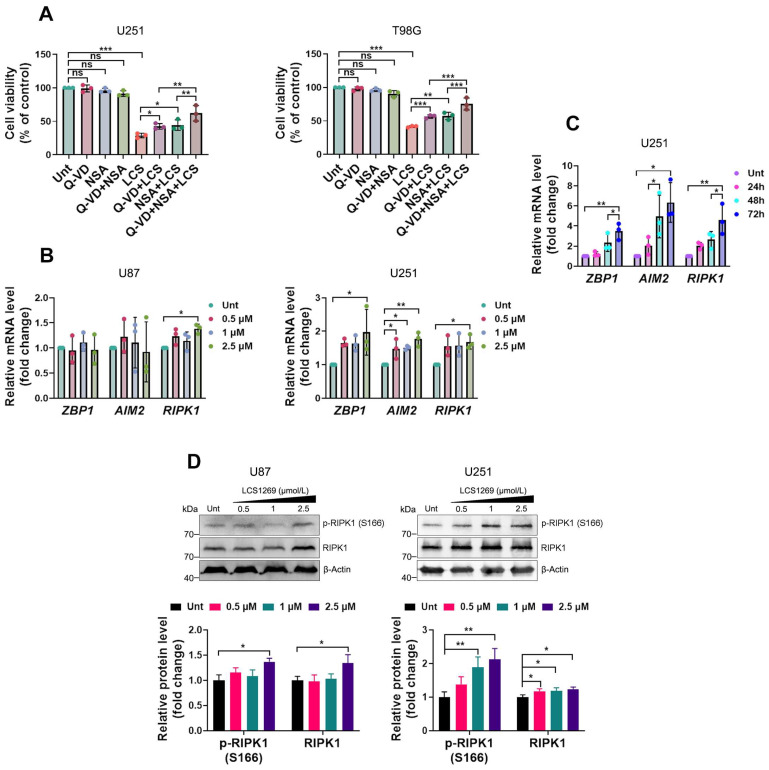
LCS1269 differentially regulates *ZBP1*, *AIM2*, and *RIPK1* gene expression in the GBM cells. (**A**) The MTT viability assay of the U251 and T98G cells that were pre-incubated for 1 h with the irreversible pan-caspase inhibitor Q-VD-OPh (25 µM), the selective MLKL inhibitor necrosulfonamide (NSA) (5 µM), or both inhibitors simultaneously, followed by the LCS1269 treatment (2.5 µM) for 72 h. (**B**) The qRT-PCR analysis of the mRNA levels of the three PANoptosome genes, *ZBP1*, *AIM2*, and *RIPK1*, in the U87 and U251 cells that were treated with or without LCS1269 at the indicated concentrations for 24 h. (**C**) The qRT-PCR analysis of the time-dependent effects of LCS1269 on *ZBP1*, *AIM2*, and *RIPK1* mRNA expression in the U251 cells. (**D**) The Western blot analysis of the protein levels of p-RIPK1 (S166) and the total RIPK1 in the U87 and U251 cells that were treated with or without LCS1269 at the indicated concentrations for 24 h (upper). The histograms show the densitometric analysis of the band intensity for specific proteins that were normalized to β-Actin (lower). The data are presented as mean ± SD from at least three independent experiments. * *p* < 0.05, ** *p* < 0.01, *** *p* < 0.001, and ns—not significant.

Taking into account that LCS1269 simultaneously triggered pyroptosis, apoptosis, and necroptosis, we hypothesized that its anti-glioblastoma effects are maintained by means of a PANoptosis induction. An MTT cell viability assay clearly demonstrated that the combined application of the pan-caspase inhibitor Q-VD-OPh and the necroptosis inhibitor NSA reversed the LCS1269-induced cytotoxicity more significantly than when each inhibitor was used individually (Figure 7A).

To explore and nuance the underlying mechanisms of LCS1269-mediated PANoptosis, we examined the expression of the key PANoptosome components, ZBP1, AIM2, and RIPK1. Interestingly, we found that LCS1269 significantly overexpressed only *RIPK1* in the U87 cells, whereas the expression of all these genes was enhanced in a dose-dependent manner in the U251 cells (Figure 7B). Furthermore, the expression of *ZBP1*, *AIM2*, and *RIPK1* was also induced in a time-dependent manner in the U251 cells, with *AIM2* and *RIPK1* exhibiting the most substantial increase (Figure 7C). In addition, our findings were additionally corroborated by the Western blotting results (Figure 7D).

Altogether, we proposed that LCS1269 could induce PANoptosis that was mediated either by the RIPK1-PANoptosome alone or by the concurrent activation of ZBP1-, AIM2-, and RIPK1-PANoptosomes.

### 2.5. PANoptosis Induced by LCS1269 May Arise from Polyploidization, Which Is Accompanied by the Formation of Micronucleated Cells with Either a Ruptured or Collapsed Nuclear Envelope

**Figure 8:**
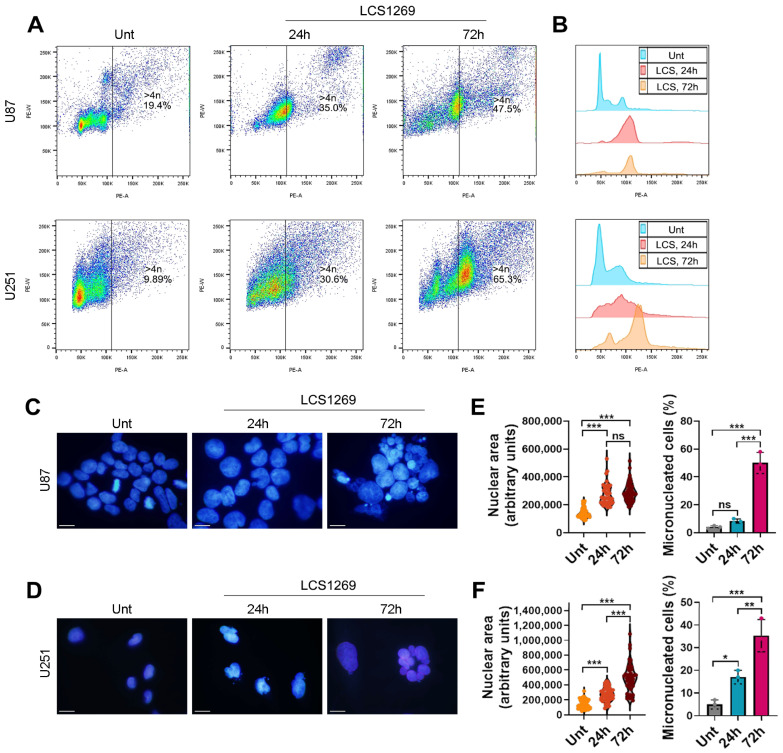
LCS1269-induced PANoptosis is coordinated with a polyplodization in the GBM cells through the formation of both micronucleated and macronucleated cells. (**A**,**B**) The representative FACS plots, gating strategy, and cell cycle alterations for the assessment of the time-course kinetics of polyploidization (cells with DNA content > 4n) in the U87 and U251 cells that were treated with or without LCS1269 (2.5 µM) for the time points as indicated, using PI staining and flow cytometry. (**C**,**D**) The representative images showing the Hoechst 33,258 staining of nuclei in the U87 and U251 cells that were exposed to LCS1269 for the indicated time points by fluorescence microscopic analysis. (**E**,**F**) The U87 and U251 cells were treated and subjected as above, and the relative nuclear area was measured by ImageJ v. 2.14.0 (National Institutes of Health, Bethesda, MD, USA). The quantity of micronucleated cells was counted by hand. A minimum of 100 cells from 5 to 10 random fields were processed (magnification, 1000×). The scale bar = 10 µm. The data are expressed as mean ± SD from at least three independent experiments. * *p* < 0.05, ** *p* < 0.01, *** *p* < 0.001, and ns—not significant.

As shown in Figure 8A,B, LCS1269 markedly augmented the polyploidization in both the U87 and U251 cells. The microscopic analysis further revealed that after a 24 h exposure to LCS1269, polyploidization in the U87 cells occurred predominantly through a macronuclei formation (Figure 8C,E). In contrast, the treatment of the U251 cells with LCS1269 not only induced the formation of macronucleated cells but also led to a significant increase in the percentage of micronucleated cells (Figure 8D,F).

**Figure 9:**
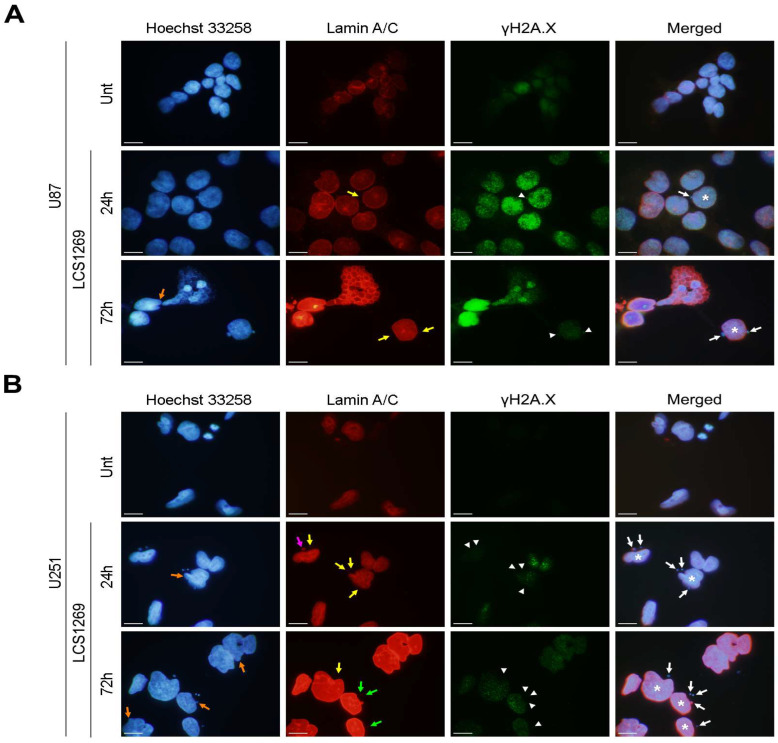
The micronuclei from the GBM cells that were treated with LCS1269 have a defective nuclear envelope (**A**,**B**) The immunofluorescence staining for lamin A/C, γH2A.X, and Hoechst 33258 in the U87 and U251 cells that were treated with or without LCS1269 (2.5 µM) for the time points as indicated (white asterisks and arrows mark the primary nuclei and micronuclei, respectively; the orange arrows indicate nuclear protrusion (“buds”); the green and yellow arrows denote the ruptured and collapsed nuclear envelope, respectively; the pink arrow shows the micronucleus with a practically intact nuclear envelope; and the white arrowheads delineate the micronuclei with a considerable decrease or absence of γH2A.X labeling). The magnification is 1000×. The scale bar = 10 µm.

Importantly, the immunofluorescence staining for lamin A/C clearly showed that micronuclei that were triggered by LCS1269 predominantly had a ruptured and collapsed nuclear envelope (green and yellow arrows, respectively). In contrast, micronuclei surrounded by a practically intact nuclear envelope (pink arrows) were overwhelmingly rare (Figure 9B). As can be seen in Figure 9, the micronuclei with an unaltered nuclear envelope, as well as those with defects such as a reduced or absent lamin A/C staining (white arrows), exhibited a marked decrease or absence (white arrowheads) of γH2A.X foci—a marker of DNA damage—compared to the primary nuclei (white asterisks). Interestingly, we revealed that the micronuclei that were induced by the LCS1269 treatment appear to result from so-called “nuclear budding” (Figure 9A, leftmost panel; orange arrows denote early and nearly fully formed nuclear buds).

Collectively, our findings suggest that the LCS1269-induced micronuclei formation and the associated nuclear envelope impairment, coupled with a deficient DNA damage response in these micronuclei, could lead to a loss of nuclear compartmentalization. This breakdown of the critical barrier between genomic DNA and cytosol may ultimately result in the induction of PANoptosis in the GBM cells.

## 3. Discussion

We have previously demonstrated that LCS1269 significantly suppressed the proliferation of both the GBM cell lines and the patient-derived GBM cultures in vitro and in vivo. This growth-inhibitory effect was attributed to a severe G2 cell cycle block associated with CDK1 activity modulation, influence on the Wee1/Myt1 pathway, and direct as well as topoisomerase I-dependent DNA damage [14,20]. Additionally, using immunocompetent mice that were treated with LCS1269 (once at a dose of 60 mg/kg for 4 h) and subsequent fluorescent microscopic analysis, we detected LCS1269 in the lumen of blood vessels of the brain (unpublished data). However, these experiments must be repeated to draw a convincing conclusion about the ability of LCS1269 to penetrate the blood–brain barrier. At the same time, these preliminary results are consistent with our published in silico ADMET prediction data [14].

In the present work, we revealed that LCS1269 drastically depolarized the mitochondrial membrane potential (MMP) and strongly augmented apoptosis in the GBM cells. This was accompanied by the downregulation of pivotal anti-apoptotic proteins such as Bcl-2, Bcl-xL, and Mcl-1. Currently, it is repeatedly documented that many natural and synthetic analogs of rebeccamycin and staurosporine, for instance, loonamycin A, EC-70124, and ICP-1, which share the indolo[2,3-*a*]pyrrolo[3,4-*c*]carbazole scaffold with LCS1269, also exert a potent cytotoxicity and induce apoptosis in diverse solid and hematological tumor cells [21,22,23,24,25]. It should be noted that our results clearly confirm that the LCS1269 treatment not only liberates the key enzymes of the intrinsic apoptosis pathway, cleaved forms of caspase-3 and caspase-7, but also causes the overexpression of several genes of the extrinsic apoptotic pathway, especially *FasL* and its receptor *Fas* (*CD95*).

Of note, a large body of evidence suggested that intrinsic and extrinsic apoptotic pathways mainly differed from each other upstream of their signal cascades, whereas the downstream molecules, executioner caspase-3, caspase-6, and caspase-7, were common for both pathways [26,27]. Thus, we proposed that LCS1269-induced apoptosis concurrently engaged both intrinsic and extrinsic pathways, ultimately converging on the proteolytic activation of the executioner caspases, particularly caspase-3 and caspase-7.

Interestingly, the combination of LCS1269 with the pan-caspase inhibitor Q-VD-OPh moderately attenuated the GBM cell death, suggesting that LCS1269 induces cell death through pathways beyond apoptosis. Specifically, our flow cytometry data, LDH leakage results, and Hoechst 33258/PI double staining assay clearly confirmed that LCS1269 may trigger one or more lytic PCD pathways.

The lytic cell death modalities, such as necroptosis, pyroptosis, and the functionally peculiar form of PCD designated as PANoptosis, are known to play essential roles in GBM progression and the outcomes of anti-glioblastoma therapy [28,29,30]. It has been shown that the necroptosis induction can be initiated by the activation of specific surface death receptors, particularly TNFR1 (by binding to TNFα) and CD95/Fas (by binding to FasL) [31,32]. The activated TNFR1 and CD95/Fas then recruit RIPK1, which, after autophosphorylation, engages with downstream RIPK3 [30,33]. Ultimately, the RIPK1-RIPK3 necroptotic pathway is executed through MLKL phosphorylation and the subsequent p-MLKL (S358)-dependent pore formation in the plasma membrane [34,35]. Our data suggested that the LCS1269 treatment killed the GBM cells by mediating MLKL-dependent necroptosis, which is evidenced by both the modulation of the p-MLKL (S358) expression and its enhanced translocation to the plasma membrane. Furthermore, the selective pharmacological inhibition of MLKL with the necrosulfonamide (NSA) significantly restored the viability of both the U251 and T98G cells, confirming that LCS1269 can stimulate necroptosis in the GBM cells.

We also reported for the first time that LCS1269 simultaneously induced another form of lytic death, pyroptosis, preferentially through the caspase-3/GSDME axis. This finding is consistent with the in-depth studies that demonstrate that the caspase-3-dependent cleavage of GSDME can promote pyroptosis in response to various cytotoxic drugs [36,37]. Moreover, our experiments involving the combined application of Q-VD-OPh and NSA inhibitors revealed that apoptosis, necroptosis, and pyroptosis that are triggered by LCS1269 did not occur as separate, individual processes. Instead, they represented a coordinated form of PCD known as PANoptosis.

Historically, apoptosis, necroptosis, and pyroptosis were considered as largely independent and non-overlapping pathways [38]. However, comprehensive studies of their molecular mechanisms have made it clear that the signaling cascades of these three PCD modes share extensive crosstalk and can even co-exist upon the induction of PANoptosis [39,40]. Indeed, using qRT-PCR and the Western blot analysis, we further confirmed that LCS1269 could provoke PANoptosis via the RIPK1-PANoptosome alone or via the integrated ZBP1-, AIM2-, and RIPK1-PANoptosomes. Notably, a classical apoptosis inducer that is structurally similar to LCS1269, staurosporine, has also been shown to drive RIPK1-mediated PANoptosis in diverse cell types [41].

Since necroptosis, pyroptosis, and PANoptosis are accompanied by the rupture of the plasma membrane and the outflow of intracellular content (mainly damage-associated molecular patterns (DAMPs), such as ATP and damaged DNA), they may promote both a passive and active inflammatory response [38]. As a result, the activation of the master regulator of many of the pro-inflammatory signaling pathways, the transcription factor NF-κB, might be induced [42,43]. As was expected, we detected an obvious upregulation of both the total and phosphorylated forms of NF-κB, which were paralleled by an enhanced translocation of NF-κB from the cytoplasm to the nucleus and an augmented transcriptional activity of NF-κB in the NF-κB-Luc reporter assay.

It is well known that PANoptosome complexes (involving ZBP1, AIM2, and RIPK1) and the NF-κB pathway overlap and are jointly implicated not only in pathogen-associated inflammatory responses but also in so-called “sterile inflammation” [44,45,46,47]. ZBP1, AIM2, and RIPK1 are crucial sensors that prime the corresponding PANoptosomes in response to self-DNA as a key DAMP [48,49]. Multiple lines of evidence have pointed out that the micronuclei frequently possess diverse defects in their nuclear envelope and thus are a source of self-DNA [50,51].

Here, we showed that the exposure of the GBM cells to LCS1269 triggered remarkable polyploidization, including a significant increase in the percentage of micronucleated cells. We also found that the expression of lamin A/C, an important component of the nuclear lamina, was greatly reduced or absent in the micronuclei of LCS1269-treated cells. Additionally, the immunofluorescence staining for γH2A.X (a DNA damage marker) clearly demonstrated that the intensity of fluorescence for this Histone H2A variant was weakened or lost in the micronuclei compared to the primary nuclei of the LCS1269-treated GBM cells. Indeed, a fundamental role for the micronuclei in the control of apoptosis, necroptosis, and pyroptosis has been thoroughly documented [52]. Likewise, the balanced lamin A/C expression as an important factor in the sensitivity of cancer cells to conventional drugs and the induction of different PCD programs has previously been described [53,54].

The results obtained allow us to conclude that the LCS1269-mediated micronuclei formation, engaging both lamin A/C-related micronuclear envelope insufficiency and the altered γH2A.X expression, underlies the disruption of the nuclear–cytoplasmic compartmentalization. This process is essential for simultaneously producing several lytic cell death programs—pyroptosis, apoptosis, and necroptosis—to coordinately initiate PANoptosis in the GBM cells.

While the U251 cell line provided a tractable model for the mechanistic studies, our conclusions are tempered by the fact that they are derived primarily from a single GBM cell line, U251, particularly regarding the plasma membrane localization of p-MLKL (S358) and the upregulation of pyroptosis-related proteins. Future studies utilizing a panel of GBM cell lines and patient-derived GBM cell cultures are necessary to confirm the translational potential of our findings.

## 4. Materials and Methods

### 4.1. Cell Lines and Reagents

The U87, U251, and T98G GBM cells, as well as the mammary gland adenocarcinoma cell line MCF-7, were purchased from ATCC (Manassas, VA, USA) and cultivated as described previously [14]. The GBM cell lines used in this study were authenticated using the COrDIS EXPERT 26 STR profiling kit (Gordiz, Moscow, Russia). All the cells were tested and found to be negative for mycoplasma contamination by a quantitative real-time PCR using the following primers for Mycoplasma spp.: 5′-GGGAGCAAACAGGATTAGATACCCT-3′ (forward), 5′-CACCATCTGTCACTCTGTTAACCTC-3′ (reverse), and/or by DAPI staining. LCS1269 was synthesized as previously reported [55]. The high-performance liquid chromatography spectra (HPLC) and the corresponding analytical data are given in Figure S2. The JC-1 (5,5′,6,6′-tetrachloro-1,1′,3,3′-tetraethylbenzimidazolocarbocyanine iodide) dye was from ThermoScientific (Waltham, MA, USA). The Hoechst 33258, propidium iodide (PI), and DAPI (4′,6-diamidino-2-phenylindole, dilactate) were received from Sigma-Aldrich (St. Louis, MO, USA). An irreversible pan-caspase inhibitor quinoline-Val-Asp-difluorophenoxymethylketone (Q-VD-OPh) was from MedChemExpress (Monmouth Junction, NJ, USA). The selective MLKL inhibitor Necrosulfonamide (NSA) was from Cayman Chemical (Ann Arbor, MI, USA). The TurboFect Transfection Reagent was purchased from ThermoScientific (Waltham, MA, USA).

### 4.2. Mitochondrial Membrane Potential (MMP) Measurements

#### 4.2.1. Microscopic Visualization

The U87, U251, and T98G (1 × 10^5^/well) cells were placed into 6-well plates (SPL, Naechon-Myeon, Republic of Korea) on coverslips overnight to attach. The next day, cells were treated with or without LCS1269 (2.5 µM) and incubated for 24 h. Then, cells were gently washed twice with PBS and stained with JC-1 (1 µg/mL) at 37 °C for 30 min, followed by an assessment of the MMP collapse using a fluorescence microscope Axioplan 2 (Carl Zeiss AG, Oberkochen, Germany) at an original magnification of 400×.

#### 4.2.2. Flow Cytometry Detection

The U87 cells grown in 6-well plates (SPL, Pocheon-si, Republic of Korea) (1 × 10^5^ per well) were incubated in the absence or presence of LCS1269 (2.5 µM) for 24, 48, and 72 h, respectively. After this, the cells were harvested by a warm Versen solution (PanEco, Moscow, Russia), washed twice with PBS, and stained with JC-1 (1 µg/mL) at 37 °C for 30 min. The MMP was subsequently monitored by a FACSCanto2 flow cytometer (BD Biosciences, Franklin Lakes, NJ, USA) and FACSDiva 6.1.3 using at least 1 × 10^4^ cells per sample.

### 4.3. Annexin V-FITC/PI Double Staining Assay

The cells (U87 and U251) that were cultivated at a density of 1 × 10^5^/well in 6-well plates (SPL, Pocheon-si, Republic of Korea) either were or were not exposed to LCS1269 (2.5 µM) for 24, 48, and 72 h, respectively. The cells were further rinsed with a warm Versen solution (PanEco, Moscow, Russia), collected using a 0.25% trypsin-EDTA solution (Paneco, Moscow, Russia), and probed with a FITC-conjugated Annexin-V antibody (BioLegend, San Diego, CA, USA) that was diluted in an Annexin-binding buffer (10 mmol/L HEPES [pH 7.4], 140 mmol/L NaCl and 2.5 mmol/L CaCl_2_) and a PI solution (1 µg/mL), and it was subsequently processed as previously described [55]. The T98G cells were pretreated with the cell-permeable pan-caspase inhibitor Q-VD-OPh (25 µmol/L) 1 h prior to the addition of LCS1269 (2.5 µM). Following an incubation of 72 h, the percentage of cells gated as live (Annexin-V^−^/PI^−^), apoptotic (Annexin-V^+^/PI^−^), late apoptotic/necroptotic/pyroptotic (Annexin-V^+^/PI^+^), and necrotic (Annexin-V^−^/PI^+^) was determined using a FACSCanto2 flow cytometer (BD Biosciences, Franklin Lakes, NJ, USA) and FACSDiva 6.1.3.

### 4.4. Quantitative Real-Time PCR (qRT-PCR)

The total RNA was prepared from LCS1269, which was treated for 24 h, and from untreated GBM cells using a TRI reagent (Molecular Research Center, Cincinnati, OH, USA). The detailed procedures for the RNA extraction, reverse transcription, and polymerase chain reaction conditions were previously described [14,55]. The tyrosine 3-monooxygenase/tryptophan 5-monooxygenase activation protein zeta (*YWHAZ*) gene expression was used to normalize the target gene mRNA expression. The 2^−ΔΔCt^ method was approved to calculate a relative gene expression [56]. The sequences of the primers used for amplification are given in Table S1.

### 4.5. Protein Lysates Preparation, SDS-PAGE, and Western Blotting

The GBM cells were treated with 0, 0.5, 1, and 2.5 µM of LCS1269 for 24 h or were exposed to 0 and 2.5 µM of LCS1269 for 24, 48, and 72 h, respectively. Then, the cells were lysed on ice by a commercial RIPA buffer (ThermoScientific, Waltham, MA, USA) supplemented with protease and phosphotase inhibitor cocktails (Roche, Basel, Switzerland), and the concentration of the total protein was measured using a bicinchoninic acid (BCA) kit (Millipore, South San Francisco, CA, USA). Finally, 30 µg of the obtained whole lysates were further separated by 7.5–12% SDS-PAGE and were analyzed utilizing Western blotting as previously described [14]. The primary antibodies that were used in this work are listed in Table S2.

### 4.6. MTT Cell Viability Assay

The GBM cells that were cultured in 96-well plates (5 × 10^3^ per well) were added to the pan-caspase inhibitors Q-VD-OPh (25 µmol/L) and NSA (5 µmol/L) individually or in combination 1 h prior to the addition of LCS1269 (2.5 µM). After an incubation of 72 h, the cells were processed as described [14].

### 4.7. Lactate Dehydrogenase (LDH) Release Assay

The GBM cells that were grown in the 96-well plates (SPL, Pocheon-si, Republic of Korea) at a density of 5 × 10^3^ per well were exposed to 0, 0.5, 1, and 2.5 µM of LCS1269 for 24, 48, and 72 h, respectively. Furthermore, 50 µL of supernatant from each well was transferred to a new 96-well plate, and the level of the LDH release was estimated using a cytotoxicity LDH assay kit (MedChemExpress, Monmouth Junction, NJ, USA) according to the manufacturer′s protocol.

### 4.8. Hoechst 33258/PI Fluorescence

The U251 and T98G cells (1 × 10^5^/well) were seeded on coverslips in 6-well plates (SPL, Pocheon-si, Republic of Korea) overnight in order to attach. Then, the cells either were or were not exposed to the presence of LCS1269 (2.5 µM) for 48 h. After this, cells were once rinsed with PBS to wash out floating cells and were stained with a working solution containing the serum-free DMEM medium, Hoechst 33258 (10 µg/mL), and PI (1 µg/mL) for 30 min. Finally, the coverslips were placed on a microscopic slide and were immediately captured by a fluorescence microscope Axioplan 2 (Carl Zeiss AG, Oberkochen, Germany) at an original magnification of 400×.

### 4.9. Luciferase Reporter Assay

The GBM cells (1 × 10^5^/well) that were cultured in 24-well plates (Corning, New York, NY, USA) were transiently transfected with a plasmid (0.25 µg/mL) containing the luciferase reporter gene controlled by tandemly repeated responsive elements to NF-κB. The NF-κB-Luc reporter plasmid used in this study was kindly provided by Dr. Alexander Gasparian [57]. A procedure of transfection was conducted in the serum-free DMEM medium at 37 °C for 5 h using a TurboFect^©^ transfection reagent (Thermo Scientific, Waltham, MA, USA) according to the manufacturer′s instructions. After this, the transfection medium was discarded, and the fresh complete DMEM was added. The next day, the cells were treated with or without LCS1269 (2.5 µM). Following an incubation of 24 h, the cells were lysed by a reporter lysis buffer (Promega, Madison, WI, USA) and further processed using a recommended protocol (Promega). To control the efficacy and toxicity of the transfection, the cells were co-transfected with the β-galactosidase plasmid. The relative reporter gene activity was measured by Tecan Infinite M200 Pro (Tecan, Männedorf, Switzerland) and calculated in arbitrary units as a ratio of detected luciferase activity to the β-galactosidase activity (β-Gal).

### 4.10. Immunofluorescence Staining

The cells (1 × 10^5^/well) were seeded on coverslips overnight to attach. Following incubation in the absence or presence of LCS1269 (2.5 µM) for 24 h, 48 h, and/or 72 h, the cells were washed once with PBS and fixed with a 4% paraformaldehyde solution for 15 min. Then, the cells were permeabilized with Triton X-100 for 5 min, rinsed thrice with PBS, blocked with 1% BSA for 15 min, and incubated with primary antibodies (NF-kB p65 [E379], Abcam (Cambridge, UK), cat.# ab32536; phospho-MLKL (Ser358) (YA16231), MedChemExpress, cat.# HY-P81878; Lamin A/C (636), Santa Cruz Biotechnology (Dallas, TX, USA), sc-7292; phospho-Histone H2A.X (Ser139) (20E3), Cell Signaling Technology (Danvers, MA, USA), cat.# 9718) for 1 h. After this, the cells were washed thrice with PBS and probed with a secondary goat anti-mouse Alexa Fluor 488 antibody (ThermoScientific, Waltham, MA, USA) or a goat anti-rabbit Alexa Fluor 488 antibody (ThermoScientific, Waltham, MA, USA) mixed with DAPI for 1 h. Finally, the cells were covered with IMMU-MOUNT (ThermoScientific, Waltham, MA, USA) and captured using a fluorescence microscope Axioplan 2 (Carl Zeiss AG, Oberkochen, Germany) at an original magnification of 1000×.

### 4.11. Polyploid Cells Assessment

The GBM cells that were cultured in 6-well plates (SPL, Pocheon-si, Republic of Korea) were incubated in the absence or presence of LCS1269 (2.5 µM) for 24 h and 72 h. Then, for flow cytometry analysis, the cells were rinsed in a warm Versen solution (PanEco, Moscow, Russia), harvested by a 0.25% trypsin-EDTA solution (Paneco, Moscow, Russia), and fixed with 70% ethanol at 4 °C for at least 3 h. After centrifugation, the cell pellet was washed once with PBS and treated with RNAse A (ThermoScientific, Waltham, MA, USA) for 30 min at 37 °C. The cells were further resuspended in 400 μL of PI solution (0.5 mg/mL in PBS) and incubated for 30 min in the dark. The DNA content distribution was determined by a FACSCanto2 flow cytometer (BD Biosciences, San Diego, CA, USA) using at least 20,000 cells per sample. The data obtained were processed utilizing FlowJo V10 software (Tristar, CA, USA).

For microscopic visualization, the cells grown on the coverslips and incubated with (2.5 µM) or without LCS1269 for 24 h and 72 h were then fixed in 4% paraformaldehyde solution for 15 min, permeabilized with Triton X-100 for 5 min, and stained with a Hoechst 33258 dye solution (10 μg/mL) for 20 min. After washing with PBS, the coverslips were covered with IMMU-MOUNT (ThermoScientific, Waltham, MA, USA) and imaged in random fields at a magnification of 1000× using an Axioplan 2 fluorescence microscope (Carl Zeiss AG, Oberkochen, Germany) and AxioVision v. 4.1 software (Carl Zeiss AG, Oberkochen, Germany).

### 4.12. Scanning Electron Microscopy (SEM)

The detailed protocol of SEM used in this study was previously described [58]. The GBM cells were viewed and captured using a SCAN Quattro S scanning electron microscope (ThermoScientific, Waltham, MA, USA), and MCF-7 cells micrographs were obtained by a JSM-6380 LA scanning electron microscope (Jeol, Tokyo, Japan). All the SEM studies were carried out at the Shared Research Facility “Electron microscopy in life sciences” at Moscow State University (Unique Equipment “Three-dimensional electron microscopy and spectroscopy”).

### 4.13. Statistical Analysis

The data were expressed as mean ± standard deviation (SD) and calculated with GraphPad Prism version 10.4.1 software (GraphPad Software, San Diego, CA, USA). Each experiment was repeated at least three times. The statistical differences were analyzed using ANOVA (one-way analysis of variance) followed by a Newman–Keuls *post hoc* test (for more than two cohorts of variables) or an unpaired *t*-test (for two groups of variables). *p* values of <0.05 (*), <0.01 (**), and <0.001 (***) were considered as statistically significant.

## 5. Conclusions

In summary, our findings reported in this study delineate a peculiar form of PCD, PANoptosis, as the cell death decision of the GBM cells in response to the LCS1269 treatment. We ascertain several mechanisms underlying both non-lytic (apoptosis) and lytic (necroptosis and pyroptosis) PCD modes that constitute the basis of LCS1269-mediated PANoptosis. We additionally show that the micronuclei formation, resulting from DNA damage-provoked genome instability (polyploidization), may play a key role in LCS1269-triggered PANoptosis. Understanding the ability of LCS1269 to induce lytic PCDs may have a broader significance for the immune response, particularly in the context of immunogenic cell death (ICD). ICD may be a substantial therapeutic approach for GBM treatment, potentiating the effects of conventional anti-glioblastoma drugs. Overall, the current study opens up new frontiers for further elucidating LCS1269 as a promising antitumor compound.

## Data Availability

The original contributions presented in this study are included in the article/Supplementary Materials. Further inquiries can be directed to the corresponding author.

## Supplementary Materials

The following supporting information can be downloaded at https://www.mdpi.com/article/10.3390/ph19040535/s1. Figure S1: Effect of LCS1269 on morphological features of pyroptosis in the constitutive caspase-3/GSDME double mutated MCF-7 cells; Figure S2: HPLC analysis of LCS1269. LCS1269 purity was 98–99%; Table S1: Primers used for quantitative real-time PCR (qRT-PCR); Table S2: Primary antibodies used in Western blot analysis.
