# Supplementary material for: Killing Glioblastoma Cells with Glycosylated Indolocarbazole-Based Derivative LCS1269: A Potential Crosstalk Between Micronuclei Formation and the Concurrent Induction of Apoptosis, Necroptosis, and Pyroptosis
Source: Pharmaceuticals (Basel). 2026 Mar 26;19(4):535. doi: 10.3390/ph19040535 (PMC13119239; doi:10.3390/ph19040535)
Supplement: Supplementary file 1 [file pharmaceuticals-19-00535-s001.zip › pharmaceuticals-4207168-supplementary.pdf]

Killing glioblastoma cells with glycosylated indolocarbazole-based derivative LCS1269: a potential crosstalk between micronuclei formation and the concurrent induction of apoptosis, necroptosis, and pyroptosis

Nikolay Kalitin [1,*], Alexander Masyutin [2], Maria Erokhina [2], Ekaterina Savchenko [3], Nadezhda Samoylenkova [3], Aida Karamysheva [1] and Galina Pavlova [3,4]

[1] Laboratory of Predictors of Sensitivity to Antitumor Therapy, N.N. Blokhin National Medical Research Center of Oncology, 115478 Moscow, Russia; aikaram@yandex.ru

[2] Department of Cell Biology and Histology, Faculty of Biology, Lomonosov Moscow State University, 119234 Moscow, Russia; imber.acidis@gmail.com (A.M.); masha.erokhina@gmail.com (M.E.)

[3] Laboratory of Molecular and Cellular Neurogenetics, N.N. Burdenko National Medical Research Center of Neurosurgery, 125047 Moscow, Russia; savhenko61@mail.ru (E.S.); samoylenkova.n@gmail.com (N.S.); lkorochkin@mail.ru (G.P.)

[4] Laboratory of Neurogenetics and Developmental Genetics, Institute of Higher Nervous Activity and Neurophysiology of RAS, 117485 Moscow, Russia

* Correspondence: f.oskolov@mail.ru

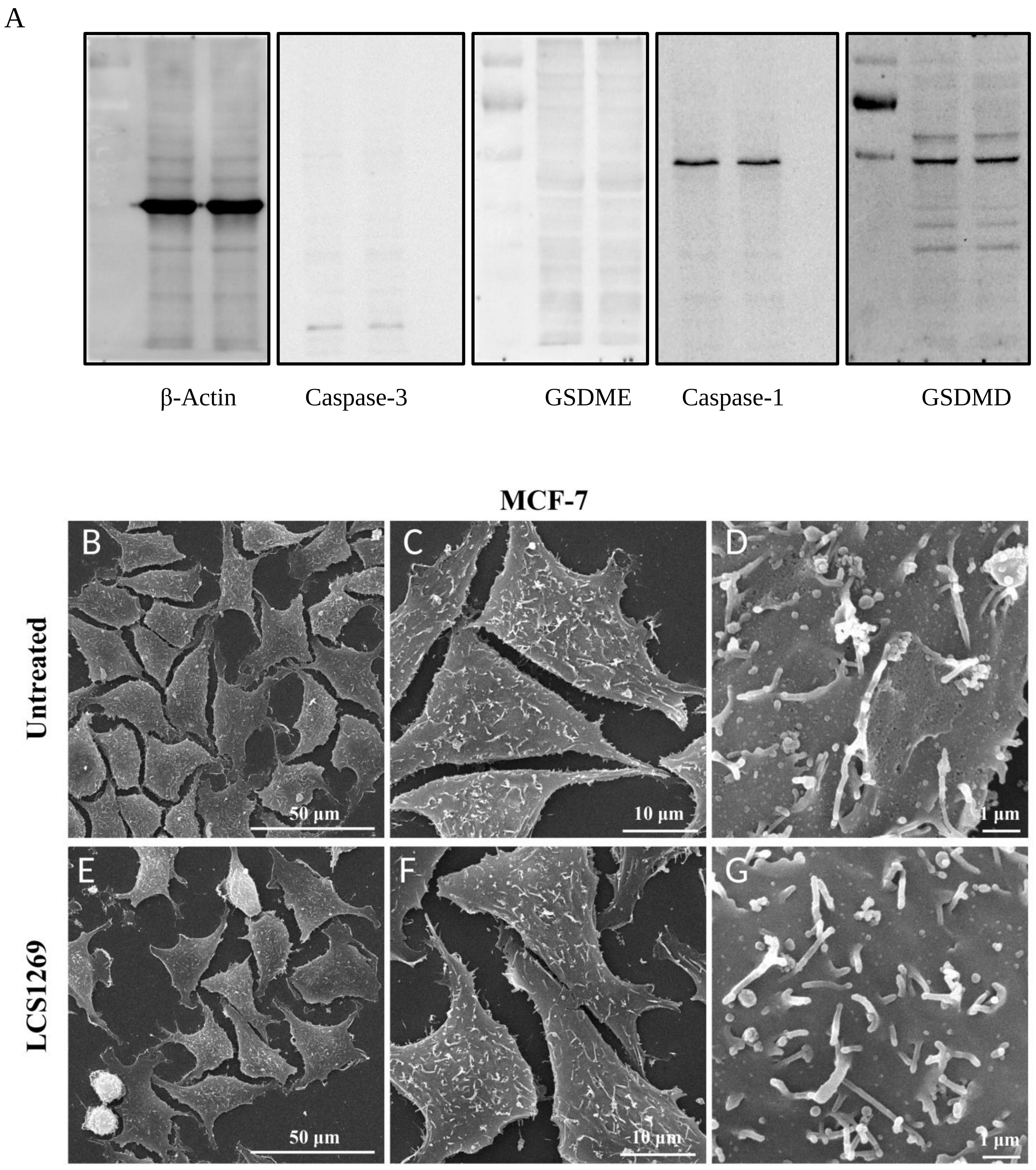

Figure S1. Effect of LCS1269 on morphological features of pyroptosis in the constitutive caspase-3/GSDME double mutated MCF-7 cells.

(**A**) Representative images showing baseline content of caspase-3, GSDME, caspase-1, and GSDMD proteins expressed in MCF-7 cells. (**B–D**) Scanning electron micrographs of untreated MCF-7 cells

captured at the different magnification. (**E–G**) Cell surface of MCF-7 cells exposed to LCS1269 (25 μM) for 48h. . β-Actin was used as a loading control.

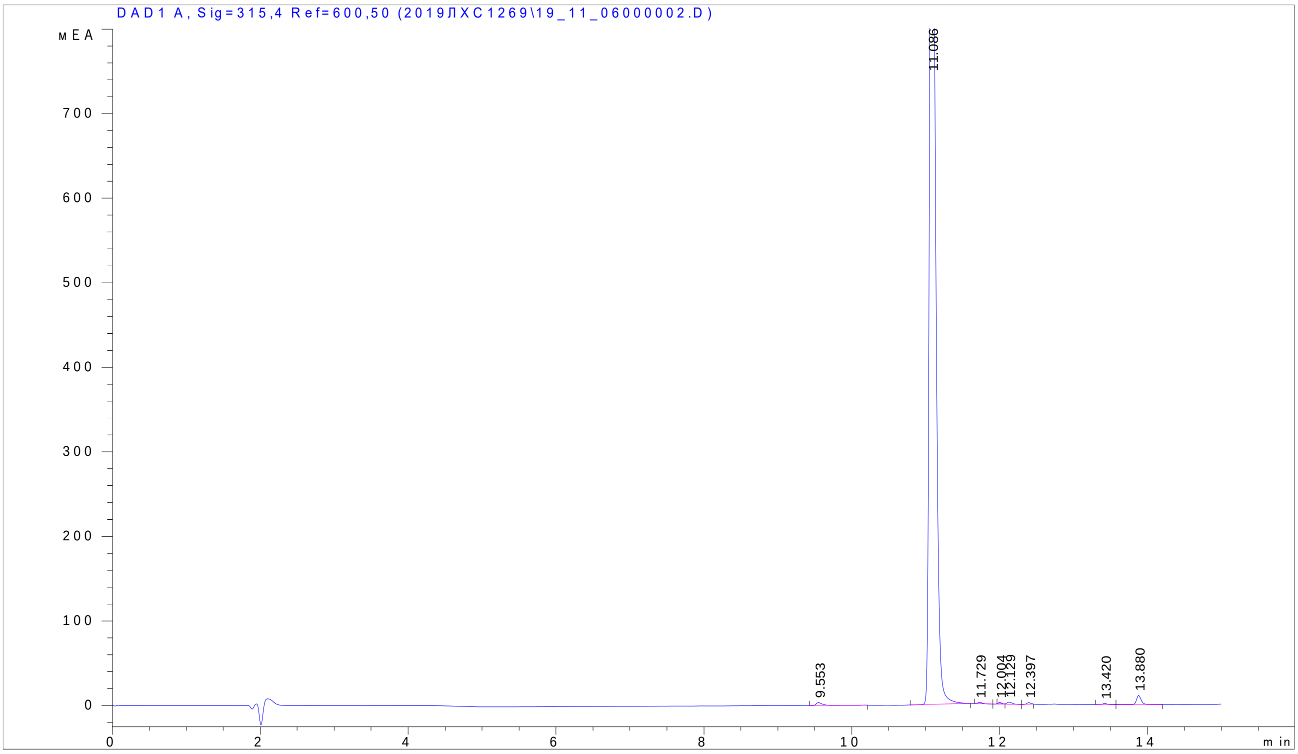

Figure S2. HPLC analysis of LCS1269. LCS1269 purity was 98 – 99%. The gradient elution at 40°C was carried out. The gradient started at water/acetonitrile (9:1) + trifluoroacetic acid 0.1% (mobile phase A) and ended at water/acetonitrile (0.5:9.5) + trifluoroacetic acid 0.1% (mobile phase B) after 15 min. The retention time was 10.9 ± 0.2 min, λ 315 nm.

Table S1. Primers used for quantitative real-time PCR (qRT-PCR)

	Gene	Primer sequences
1	*TNF-α*	5′-GAGGCCAAGCCCTGGTATG-3′ (forward)
		5′-CCGGCCGATTGATCTCAGC-3′ (reverse)
2	*FasL*	5′-TGCCTTGGTAGGATTGGGC-3′ (forward)
		5′-GCTGGTAGACTCTCGGAGTTC-3′ (reverse)
3	*TRAIL*	5′-TGCGTGCTGATCGTGATCTTC-3′ (forward)
		5′-GCTCGTTGGTAAAGTACACGTA-3′ (reverse)
4	*Fas (CD95)*	5′-AGATTGTGTGATGAAGGACATGG-3′ (forward)
		5′-TGTTGCTGGTGAGTGTGCATT-3′ (reverse)
5	*TNFR1*	5′-TCACCGCTTCAGAAAACCACC-3′ (forward)
		5′-GGTCCACTGTGCAAGAAGAGA-3′ (reverse)
6	*ZBP1*	5′-AACATGCAGCTACAATTCCAGA-3′ (forward)
		5′-AGTCTCGGTTCACATCTTTTGC-3′ (reverse)
7	*AIM2*	5′-TGGCAAAACGTCTTCAGGAGG-3′ (forward)
		5′- AGCTTGACTTAGTGGCTTTGG-3′ (reverse)
8	*RIPK1*	5′-GGGAAGGTGTCTCTGTGTTTC-3′ (forward)
		5′- CCTCGTTGTGCTCAATGCAG-3′ (reverse)
9	*YWHAZ*	5′-ACTTTTGGTACATTGTGGCTTCAA-3′ (forward)
		5′-CCGCCAGGACAAACCAGTAT-3′ (reverse)

Table S2. Primary antibodies used in Western blot analysis

Antibody name	Manufacturer	Catalogue number	Dilution rate
Bcl-2 (D55G8) Rabbit mAb	Cell Signaling Technology	4223	1:1000
Phospho-Bcl-2 (Ser70) (5H2) Rabbit mAb	Cell Signaling Technology	2827	1:1000
Phospho-Bcl-2 (Thr56) Antibody	Cell Signaling Technology	2875	1:1000
Bcl-xL (54H6) Rabbit mAb	Cell Signaling Technology	2764	1:1000
Mcl-1 (D35A5) Rabbit mAb	Cell Signaling Technology	5453	1:1000
PARP Antibody	Cell Signaling Technology	9542	1:1000
Caspase-3 (D3R6Y) Rabbit Monoclonal Antibody	Cell Signaling Technology	14220	1:1000
Caspase-7 (D2Q3L) Rabbit Monoclonal Antibody	Cell Signaling Technology	12827	1:1000
Anti-NF-kB p65 antibody [E379]	Abcam	ab32536	1:1000
Phospho-NF-kappaB p65 (Ser536) (93H1) Rabbit Monoclonal Antibody	Cell Signaling Technology	3033	1:1000
MLKL Antibody	MedChemExpress	HY-P80227	1:1000

Phospho-MLKL (Ser358) Antibody (YA16231)	MedChemExpress	HY-P81878	1:500
NLRP3 Antibody	Affinity Biosciences	DF7438	1:500
Polyclonal Antibody to Caspase 1 (CASP1)	Cloud-Clone	PAB592Hu01	1:400
Polyclonal Antibody to Interleukin 1 Beta (IL1b)	Cloud-Clone	PAA563Hu01	1:1000
GSDMD Antibody	Affinity Biosciences	AF4012	1:1000
GSDME Antibody	MedChemExpress	HY-P80697	1:500
RIP (D94C12) XP® Rabbit mAb	Cell Signaling Technology	3493	1:1000
Phospho-RIP (Ser166) (D1L3S) Rabbit mAb	Cell Signaling Technology	65746	1:1000
beta-Actin (C4) Mouse mAb HRP	Santa Cruz Biotechnology	sc-47778 HRP	1:500
